# A cross-sectional study of traditional Chinese medicine practitioner’s knowledge, treatment strategies and integration of practice of chronic pelvic pain in women

**DOI:** 10.1186/s12906-021-03355-6

**Published:** 2021-06-24

**Authors:** Susan Arentz, Caroline Smith, Rebecca Redmond, Jason Abbott, Mike Armour

**Affiliations:** 1 NICM Health Research Institute, Western Sydney University, Locked Bag 1797, Penrith, NSW 2751 Australia; 2 Translational Health Research Institute, Western Sydney University, Locked Bag 1797, Penrith, NSW 2751 Australia; 3 Australian Research Centre in Complementary and Integrative Medicine, Faculty of Health, University of Technology Sydney, Ultimo, NSW Australia; 4 Office of Research, Endeavour College of Natural Health, Fortitude Valley, QLD Australia; 5 School of Women’s and Children’s Health, University of New South Wales, Barker Street, Randwick, NSW 2031 Australia

**Keywords:** Persistent pelvic pain, Vaginismus, Dysmenorrhea, Vulvodynia, Endometriosis, Dyspareunia, Acupuncture

## Abstract

**Background:**

Chronic pelvic pain (CPP) in women is persistent, intermittent cyclical and non-cyclical lower abdominal pain, lasting for more than 6 months. Traditional Chinese Medicine (TCM) is a popular treatment option for women’s health conditions, but little is known about how treatment for CPP is delivered by TCM practitioners. The aim of this survey was to explore practitioners understanding and treatment of women with CPP, and how they integrate their management and care into the health care system.

**Method:**

An online cross-sectional survey of registered TCM practitioners in Australia and New Zealand between May and October 2018. Survey domains included treatment characteristics (e.g. frequency), evaluation of treatment efficacy, referral networks, and sources of information that informed clinical decision making.

**Results:**

One hundred and twenty-two registered TCM practitioners responded to this survey, 91.7% reported regular treatment of women with CPP. Treatment decisions were most-often guided by a combination of biomedical and TCM diagnosis (77.6%), and once per week was the most common treatment frequency (66.7%) for acupuncture. Meditation (63.7%) and dietary changes (57.8%) were other commonly used approaches to management.

The effectiveness of treatment was assessed using multiple approaches, most commonly pain scales, (such as the numeric rating scale) and any change in use of analgesic medications. Limitations to TCM treatment were reported by over three quarters (83.7%) of practitioners, most commonly due to cost (56.5%) and inconvenience (40.2%) rather than safety or lack of efficacy. Sources informing practice were most often Integration within the wider healthcare system was common with over two thirds (67.9%) receiving referrals from health care providers.

**Conclusion:**

TCM practitioners seeing women with various CPP symptoms, commonly incorporate both traditional and modern diagnostic methods to inform their treatment plan, monitor treatment progress using commonly accepted approaches and measures and often as a part of multidisciplinary healthcare for women with CPP.

**Supplementary Information:**

The online version contains supplementary material available at 10.1186/s12906-021-03355-6.

## Introduction

Chronic pelvic pain (CPP) in women is defined as intermittent and continuous, cyclical and non-cyclical lower abdominal pain, lasting for more than 6 months [1, 2]. It is characterised by diverse pain symptoms including dysmenorrhea, dyspareunia, dyschezia and dysuria as well as considerable fatigue and negative impacts on mental health [3, 4]. Women with CPP are often encumbered with a substantial, physical, psychological, emotional, social and economic burden [5–8].

CPP prevalence in women worldwide ranges between 2.1 to 81% [9, 10]. In Australia, it is estimated to affect approximately 21.5% of reproductive aged women [10]. CPP represents 3.8% of primary care presentations, [11] and up to 10% of outpatient referrals to gynecologists [12]. Endometriosis and vulvodynia are two of the most commonly diagnosed causes of CPP in women of reproductive age, with estimated lifetime prevalence rates for endometriosis of approximately 11% in Australia [13] and 8–16% for vulvodynia, [14, 15]. Other conditions that may cause CPP symptoms include painful bladder syndrome, fibroids, chronic urinary tract infection, irritable bowel syndrome, inflammatory bowel disease and malignancy, as well as injuries related to childbirth, neurological entrapment and psychological and psychosocial factors [1]. CPP, regardless of cause, is significantly associated with an increased risk of psychological morbidity [16] and significantly lowered quality of life [5, 8]. Women may be negatively impacted in several aspects of their lives including employment, friendships, sexual and romantic relationships, academic study and social activities [5, 8]. Clinical guidelines recommend specific treatments for improving women’s functional ability, [17] however many treatments have limited effectiveness for reducing pain symptoms, [18] which is often a primary unmet health care need of women with diagnosed endometriosis [19]. Women with CPP due to endometriosis often report effective pain relief following surgical excision of endometriosis however surgical excision is costly and recurrence rates of pain are high with 50% of women reporting recurrence at 5 years post-surgery [18, 20]. Between 50 to 75% of women with CPP report discontinuing pharmaceutical pain management due to adverse side-effects and often explore other forms of treatment including healthcare professions from outside of the dominant biomedicine system [6, 21, 22].

Traditional, complementary and integrative medicine (TCIM) is used by 51% of women with CPP [23, 24]. Traditional Chinese Medicine (TCM) including acupuncture [24, 25] and Chinese herbal medicine [25] are popular TCIM treatments for which there is preliminary, but promising evidence of effectiveness for CPP pain reduction [26, 27]. However, despite women’s self-directed approach to care and utilisation of TCIM, referrals between medical doctors and TCM practitioners in Australia and New Zealand are often low, [28] and impeded by limited interprofessional communication [29]. Part of this may be due to the differences between health care frameworks including the holistic view of the TCM theoretical paradigm and the connected and inseparable body, mind and emotions in contrast to the more Cartesian thinking amongst biomedicine, which tends to view the body as a collection of mechanistic interactions and emphasises mind–body duality [30]. TCM practitioners have historically identified conflict between these two theoretical frameworks, with TCM being “largely incompatible” with the mechanistic framework that underpins biomedicine [31]. Therefore, it is currently unclear what role, if any, biomedical diagnosis and outcome evaluations play when TCM practitioners are treating women with CPP, and how they integrate their treatment as part of the larger, predominantly biomedical healthcare systems in Australia and New Zealand. Given the limited evidence on this topic, this study aims to explore TCM practitioner knowledge and the clinical approach to managing women with CPP and integration of TCM clinical practice in Australia and New Zealand.

## Method

### Setting

The study presents a cross-sectional survey of TCM practitioners within Australia and New Zealand. Participants were recruited through three professional associations; the Australian Acupuncture and Chinese Medicine Association (AACMA), the Federation of Chinese Medicine and Acupuncture (FCMA), and Acupuncture New Zealand. Registered members of the associations were emailed an invitation to participate between June and September 2018. Interested participants were provided with a participant information sheet, before recruitment commenced, that outlined the anonymity of the survey and implied consent at end-submission. Reminder invitations were sent via the associations in June, August, and September in 2018. The survey was conducted via web-based QualtricsXM [32] and opened for data collection in May 2018 and closed in October 2018.

### Participants

Participants were eligible for inclusion if they were registered with one of the above associations and self-identified as managing women’s health in their clinical practice. Additional inclusion criteria included English language skills and access to an internet connected device to complete the survey.

### Survey instrument

The survey was a self-administered questionnaire uploaded into the cloud-based survey administration platform Qualtrics XM [32]. Thirty three items were designed to describe TCM practitioner’s practice characteristics and their management of women with CPP (Supplementary File 1). Practice domains included practitioner’s understandings and definitions of CPP, sources of clinical information, types of interventions used, treatment patterns, interdisciplinary referrals and communication, methods used to evaluate efficacy and adverse effects, and practitioner perceived barriers to care.

The first item sought information about the proportion of women with CPP attending the practice for treatment. Subsequent items sought information about the practice characteristics including signs and symptoms of women presenting with CPP, the types and frequency of treatments for CPP and associated symptoms, practitioner perceived effectiveness, frequency of adverse effects including negative interactions with pharmaceutical treatments. Frequency of practitioner’s review of treatment effects was reported on an eleven-point Likert scale ranging from every week to once per year and/or every menstrual cycle to every third menstrual cycle. Number of treatments required to reduce CPP or associated symptoms was reported on a seven-point Likert scale ranging between 1 and 3 treatments up to more than 20 treatments, and with two options to report ‘treatment rarely reduced pain’ or ‘did not reduce pain’. Further items sought information about interdisciplinary referral networks and sources of information about CPP and treatment decisions. Frequency of interprofessional referrals during the previous eight weeks were reported on a four-point Likert scale ranging from zero to seven or more. Socio demographic characteristics and geographical location of practice were also sought. Multiple response options were available for most items to capture all information. The questionnaire took 15–20 min to complete. The questionnaire was tested for logic and readability by piloting with three Chinese medicine (CM) practitioners and edited in response to their feedback to improve question clarity.

### Ethics approval

The study was approved by the Western Sydney Human Research ethics Committee (EC00314) H12527 on the 24th of January 2018 and the Endeavour Human Research Ethics Committee (EC00358) #20180212 on the 12th of February 2018.

### Data analyses

Data were exported from Qualtrics into Microsoft 365 Excel (version 16.0) for data cleaning and statistical analysis. Responses from participants that did not treat women but completed the survey were removed. Binary and categorical variables were generated as per the survey questions for descriptive analysis. Descriptive statistics were reported using proportions and percentages. Responses to ‘other’ were reported narratively.

## Results

### TCM practitioner socio-demographics

**Fig. 1:**
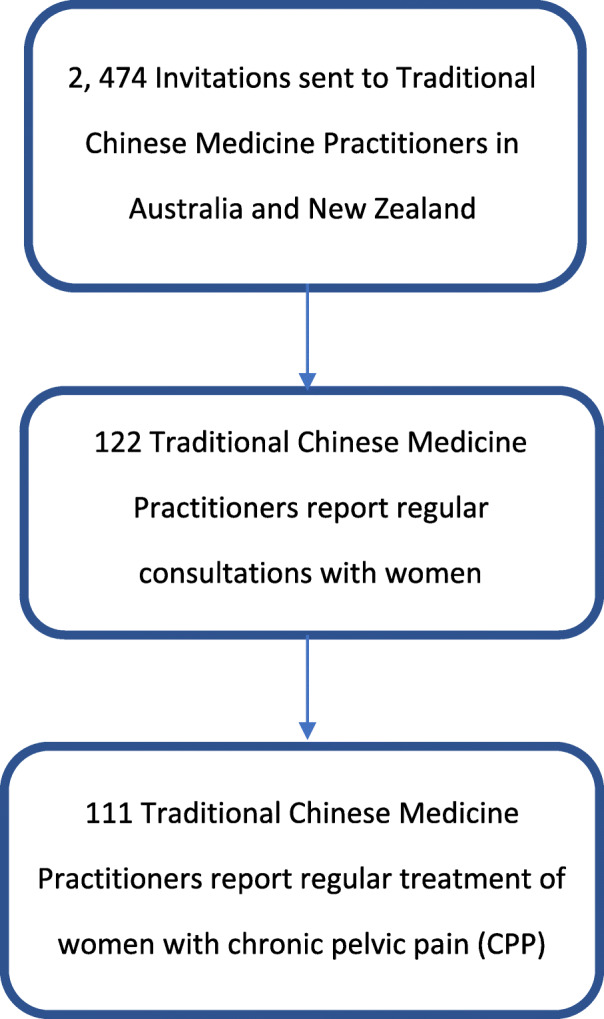
Self-reported Traditional Chinese Medicine Practitioners treatment of women with chronic pelvic pain

Two thousand four hundred and seventy-four registered practitioners were invited to complete the survey. One hundred twenty-two participants responded and reported regularly consulting with women, of which 111 practitioners (91.7%) reported they regularly treated women with CPP in their clinical practice (overall response rate 4.9%). (Fig. 1) Twenty-one (18.9%) participants reported treating a woman with CPP at least once every day they were in clinic. Most practitioners were women aged over 40 years (*n* = 53, 47.7%), in clinical practice for over 15 years (*n* = 52, 16%) and practicing 4–5 days per week (*n* = 38, 34.2%). (Table 1).

**Table 1:** Demographic characteristics of Traditional Chinese Medicine practitioners treating women with CPP

***n*** = 111	N	%
**Age**		
18–24 years	0	0
18–30 years	5	4.5
30–40 years	20	18.0
Over 40 years	53	47.7
No Answer (Missing data)	33	29.7
**Gender**		
Female	52	46.8
Male	25	22.5
Prefer to not disclose	1	0.9
No answer (Missing data)	33	29.7
**Practice characteristics**		
Employed with salary	6	5.4
Self-employed in own business	72	64.9
Full-time student	6	5.4
Informal practice (friends & family)	1	0.9
Not practicing at present	1	0.9
Other (teaching)	1	0.9
No answer (Missing data)	33	29.7
**Number of days per week in clinical practice**		
1 day per week	6	5.4
2–3 days per week	25	22.5
4–5 days per week	38	34.2
6–7 days per week	9	8.1
No answer (Missing data)	33	29.7
**Years in practice**		
Less than 1 year	2	1.8
1–3 years	6	5.4
3–6 years	8	7.2
6–10 years	15	13.5
10–15 years	15	16.0
more than 15 years	52	46.8
No answer (Missing data)	33	29.7
**Location of clinic**		
Urban	52	46.8
Regional	15	13.5
Rural	0	0
No answer (Missing data)	44	39.6
**Country of undergraduate qualification**		
Australia	60	54.1
New Zealand	5	4.5
China	6	5.4
UK or US	7	6.3
No answer (Missing data)	33	29.7

### Women’s chronic pelvic pain symptoms

Some key symptoms related to CPP, including dysmenorrhoea (*n* = 58, 52.3%) and pelvic pain related low quality of life or increased absenteeism (*n* = 37, 33.3%) were regularly treated, however some common CPP symptoms were not often treated including dyspareunia (*n* = 15, 13.5%) and dyschezia (*n* = 12, 10.8%). (Table 2). All TCM practitioners reported that at least one in four women with CPP presented an abnormal menstrual pattern according to TCM principles which was defined as an imbalance between yin and yang, temperature irregularities and by stagnation or insufficient life energy or Qi. The severity of pain was mostly assessed by questions during the consultation (*n* = 72, 64.9%), and about two thirds (*n* = 67, 60.4%) used a scale to assess the severity of pain and over half (*n* = 62, 55.8%) asked about the amount of pharmaceutical analgesia frequently used. Over half (*n* = 62, 55.9%) assessed women presenting with CPP using traditional Chinese medicine characteristics of pulse and tongue.

**Table 2:** Symptoms of women with CPP presenting to Traditional Chinese Medicine practitioners

Number of women with CPP presenting with this sign or symptom (*N* = 111)	Dyschezia/ Dysuria N (%)	Dyspareunia N (%)	Dysmenorrhoea N (%)	Absenteeism due to pain N (%)	Abnormal menstrual cycle* N (%)
All	0 (0)	0 (0)	0 (0)	0 (0)	2 (1.8)
At least 3 of 4	0 (0)	0 (0)	11 (12.2)	5 (4.5)	17 (15.3)
Over half	2 (1.8)	2 (1.8)	22 (24.4)	12 (10.8)	29 (26.1)
1–2 of 4	10 (9.0)	13 (11.7)	25 (27.8)	20 (18.0)	23 (20.7)
Less than 1 in 4	79 (71.2)	77 (69.4)	32 (35.6)	55 (49.5)	21 (18.9)
No response (missing data)	20 (18.0)	19 (17.1)	21 (18.9)	19 (17.1)	19 (17.1)

*In TCM the menstrual cycle is determined by a range of clinical features including the regularity of menstrual periods, menstrual period duration, characteristics of the pulse and tongue and by the colour and consistency of menstrual blood to assess the yin and yang balance, temperature regulation and life-force (Qi) of individuals

TCM practitioners reported potential pathological causes of CPP in women they saw in clinic as endometriosis (*n* = 74, 66.7%), fibroids (*n* = 66, 62.2%), inflammatory bowel disease (*n* = 63, 53.2%), polycystic ovary syndrome (*n* = 59, 53.2%), urinary tract infections (*n* = 59, 53.2%) and structural disorders of the lower back (*n* = 55, 49.5%). Eighteen participants (16.2%) reported other causes including interstitial cystitis, pudendal neuralgia, adhesions from surgery and due to adverse effects of medically assisted reproductive technologies (ART). Women with CPP were directly referred for biomedical pathology tests ‘often’ by 13.5%, (*n* = 15), ‘sometimes’ by 10.8%, (*n* = 12), but ‘never’ by 38.7% (*n* = 43) of TCM practitioners.

### TCM approach to chronic pelvic pain case management

A combination of biomedical and TCM diagnosis most often guided treatment decisions (*n* = 62, 55.8%), and 17.1% (*n* = 19) relied only on TCM principles. Sources guiding treatment were most often text-books and lecture notes (*n* = 38, 34.2%), followed by peer reviewed academic articles (*n* = 31, 27.9%), updates published on-line (*n* = 29, 26.1%) and discussion with clinical peers (*n* = 25, 22.5%). Other guiding treatment sources included Facebook posts, classic texts and hair tissue analyses of heavy metal concentration reported by seven (6.3%) TCM practitioners.

Pathological mechanisms understood to contribute to CPP in women included hyper-inflammation (*n* = 61, 60.4%) and muscle spasm (*n* = 44, 39.6%). One respondent cited oestrogen dominance as the main underlying mechanism of CPP in women. TCM patterns commonly reported included blood stasis (*n* = 76, 68.5%), Qi stagnation (*n* = 65, 58.6%), cold stagnation (*n* = 70, 63.1%), yang deficiency (*n* = 53, 47.7%) and damp phlegm (*n* = 57, 51.4%). Just over 7 % (*n* = 21, 7.2%) reported other TCM patterns associated with CPP including disturbance of the Shen, damp heat in the lower Jiao, and cold damp liver and spleen.

Sources of information about women with CPP included TCM texts (*n* = 56, 50.5%), short seminars (*n* = 54, 48.6%), professional association seminars (*n* = 54, 44.1%), on-line courses (*n* = 41, 36.9%) and TCM mentors and teachers (*n* = 44, 39.6%). Western medicine information sources informed CPP understanding by 36.0% (*n* = 40) and 41.4% (*n* = 46) referred to articles in peer reviewed literature. Eight percent (*n* = 9) reported referring to other sources including websites of women’s health centres of excellence (for example Jean Hailes) and social media forums, such as collegial Facebook groups.

**Fig. 2:**
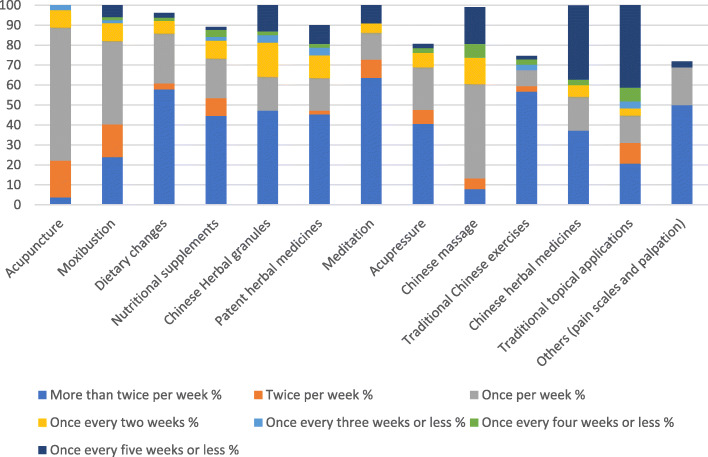
Frequency of treatment provided by TCM practitioners to women with CPP (*n* = 111)

Acupuncture was the most frequently used treatment modality (*n* = 81, 73.0%), provided once per week (*n* = 54, 48.7%) followed by moxibustion (*n* = 67, 60.4%), dietary changes (*n* = 64, 57.6%), nutritional supplements (*n* = 56, 50.5%), Chinese herbal medicine (granules (*n* = 53, 47.7%), patent herbal medicines (*n* = 53, 47.7%) and raw herbs (*n* = 35, 31.5%)), meditation (*n* = 44, 39.6%), and Chinese exercises (*n* = 37, 33.3%). Chinese massage was used by 34.2% (*n* = 38) of practitioners. (Fig. 2).

### TCM treatment effectiveness

**Fig. 3:**
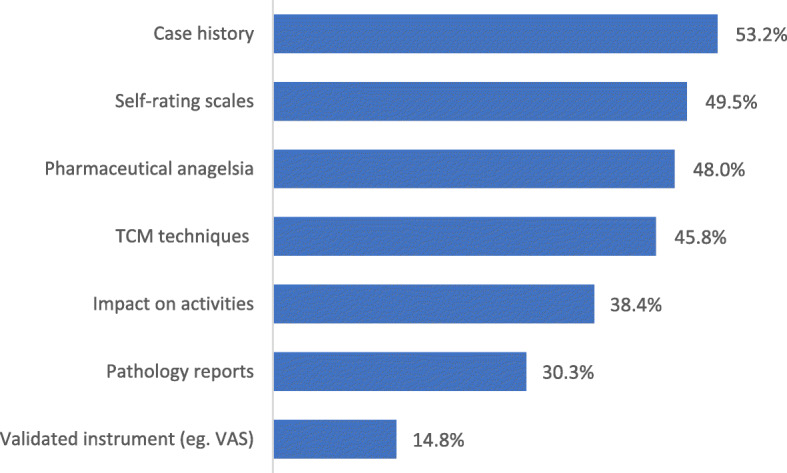
Assessments used to measure treatment effectiveness (*n* = 111)

Over 70% (*n* = 79, 71.2%) of TCM practitioners reported they perceived their treatment was effective in managing pain associated with CPP, following up to 12 treatments (*n* = 81, 73.0%). Various approaches for assessing treatment effectiveness were utilized as outlined in Fig. 3. Women’s pain was assessed during case consultation and history note taking (*n* = 59, 53.2%) and measured either using patient-reported pain scales (*n* = 55, 49.5%) and/or through the type and quantity of analgesic medication needed to control pain (*n* = 53, 47.7%). TCM diagnostic techniques (including diagnosis of tongue and pulse characteristics) were used by 51 (45.9%) practitioners to evaluate the progress of treatment. Other methods of evaluating efficacy included the use of visual analogue scales and abdominal palpation. The least reported assessment tool for pain was the use of validated instruments (*n* = 16, 14.8%). Treatment efficacy was reviewed every menstrual cycle month by a quarter of respondents (*n* = 28, 25.2%).

### Treatment limitations and adverse events

Practitioners’ perceptions of limitations and barriers to TCM treatment of women with CPP were reported by 93 (83.7%) practitioners, most often due to financial expense (*n* = 52, 46.8%) and the inconvenience (*n* = 37, 33.3%) of frequent clinical treatments (*n* = 37, 33.3%). Limited evidence of effectiveness for treatment was cited as a barrier to treatment by 27 (24.3%) and adverse effects of treatment we reported by 12.6%, (*n* = 14) of practitioners. The most reported adverse effects were worsening intensity of pain, or pain occurring at additional menstrual cycle phases, such as mid-cycle. Adverse effects were most often associated with acupuncture treatment (*n* = 11, 9.9%), including a reported serious adverse event of bowel obstruction.

### Interdisciplinary referrals and communications

Integration within the wider healthcare system was common with half (*n* = 55, 49.5%) reporting referrals from other health practitioners including 12.4% (*n* = 14) receiving seven or more referrals in the previous two weeks. General Practitioners (GPs) were the most commonly referring practitioners (*n* = 29,29.1%), followed by osteopaths (*n* = 25, 22.5%), physiotherapists (*n* = 22, 19.8%), massage therapists (*n* = 21, 18.9%) and naturopaths (m = 18, 16.2%). Western biomedical practitioner’s referrers to TCM included gynaecologists (*n* = 8, 6.3%), pelvic physiotherapists (*n* = 7, 6.3%), exercise physiologists (*n* = 6, 5.4%) and pharmacists (*n* = 4, 3.6%). Background letters of introduction were sometimes provided by referring of practitioners (*n* = 24, 21.6%), however 18.0% (*n* = 20) of TCM practitioners reported never receiving background letters for referred women with CPP.. Only one practitioner reported regular receipt of written introductions from referring practitioners.

Over half (*n* = 59, 53.2%) of TCM practitioners reported regularly referring women with CPP to other practitioners, including five (4.5%) referring over seven times in the previous two weeks. Referrals were most often to western biomedical providers including GPs (*n* = 33, 29.7%), gynaecologists (*n* = 20, 18.0%), pelvic (*n* = 11, 9.9%) and general physiotherapists (*n* = 10, 9.0%) and exercise physiologists (*n* = 8, 7.2%). Referrals to other TCIM practitioners were also common and included osteopaths (*n* = 25, 22.5%), chiropractors (*n* = 19, 17.1%), massage therapists (*n* = 16, 14.4%) and naturopaths (*n* = 11, 9.9%). Letters of introduction were reported as ‘always’ being provided by eleven (9.9%) TCM practitioners, ‘sometimes’ by 20 (18.0%) and ‘never’ by 24 (21.6%) TCM practitioners. Nearly one in five TCM practitioners (*n* = 20, 18%) reported never referring women with CPP to other health or medical practitioners.

## Discussion

This study provides insight into how TCM practitioners manage women presenting to them with CPP. Pelvic pain symptoms such as dyspareunia was an uncommon presenting symptom despite affecting almost three quarters of women with CPP [33] and may reflect the normalization of pelvic pain associated with the menstrual cycle [34] and/or TCM practitioners overlooking signs and symptoms associated with CPP. Just under two thirds of Australian women with CPP do not exclusively pursue or continue with medical care [10, 35]. They are motivated to use self-help measures and (self-perceived) low risk, natural interventions including TCIM practitioners, whose practices are often based on holistic philosophies, and may provide whole person alternatives and adjuncts to Western biomedical management, which is an expressed need of women with CPP [36]. The majority of TCM practitioners provided treatment informed by both biomedical and TCM sources. Most practitioners in this study perceived their treatments were effective however few reported evaluating the efficacy of treatments using validated methods.

TCM practitioners in our sample did not commonly use peer reviewed academic articles as part of their clinical practice, similar to previous research showing that clinical trial results do not always change practice for acupuncture practitioners [37, 38]. This may be due to practitioners’ perceptions that randomised clinical trials are not relevant to acupuncture practice, [39, 40]. However, negative attitudes towards acupuncture cited in clinical practice guidelines for CPP, may also distort TCM practitioners’ attitudes towards evidence-based practice. Derogatory reference to female users of acupuncture as ‘desperate’, [1] not only diminishes women with CPP using TCM, it describes a negative prejudice as the quality of evidence was similarly low for some medical treatments as it was for acupuncture, and yet medical users were not described as desperate. A negative prejudice in clinical guidelines may devalue TCM practitioners perceptions of evidence based clinical practice. There are a number of other factors that can impede translation of clinical trial results into community health practices, including financial and time barriers [41], similar to that reported by practitioners in this survey. This is a concern as the total number of treatments given might be an important factor in achievement of therapeutic outcomes [42–44], as practitioners may be prevented from delivering optimal ‘doses’ of acupuncture treatment [45].

Efficacy and safety has been previously demonstrated for acupuncture in the reducing pain associated with endometriosis [46, 47] and improving quality of life, [48, 49] with these pain improvements being clinically relevant as they exceed the minimally important difference for treatment of pain in women with endometriosis (at least 10 mm on a 100 mm visual analogue scale, or a 20% in absolute pain reduction) [50–52]. Whilst over two thirds of practitioners were using scales to assess the severity of women pain on presentation, none reported knowledge of how much reduction constitutes a minimal important clinical effect. The absence of a validated measure of treatment efficacy may reflect TCM lower value of research based knowledge and information over traditional techniques, [53] which is not unique to TCM but is critical in the integration of health services and interprofessional communication of referral networks.

Many practitioners reported non-integration through referral pathways into health care settings. Barriers to interprofessional referrals have been cited as being due to biomedical dominance and a lack of clarity about each other’s roles [54–56]. Cross-professional education and training about practices, mutual understanding of responsibilities and limitations, and processes including formal correspondence may assist overcoming these barriers, which is important because failures in interprofessional communication are a leading cause of patient harm [29, 54, 56]. As TCM represents 11% of primary care capability in rural areas of Australia [57] and acupuncturists up to 8.8% of services for women with other reproductive needs, [58] improved integration and shared care could improve safety as well as continuity of care and the healthcare experiences of women with CPP. TCM as part of multidisciplinary clinical care for women with CPP due to endometriosis, has been shown to improve women’s self-efficacy by cultivating confidence and resilience, relieving social isolation and improving quality of life [59].

### Limitations

Limitations of this research included low response and completion rates which reduce the validity and generalizability of the findings. Reliance on professional associations distribution of the survey limited the opportunity for reminder emails and there were no financial incentives for participants. Women’s health is not a recognized specialization for TCM practitioners in Australia or New Zealand, in contrast to other therapies such as physiotherapy, and therefore there is no way of targeting this particular cohort or ascertaining an accurate response rate given the number of eligible practitioners is unknown. Surveys of health practitioners have notoriously low response rates [60] and the response rate, and total number of responses in this survey are in line with previous data collected in the Australian and New Zealand setting [25]. Given these factors the findings are generalizable to the specific sub-group/sub-specialization of TCM practitioners who regularly treat women with CPP. The survey instrument was cross-sectional and designed to explore the subjective, self-reported opinions of TCM practitioners in their clinical approach to women presenting with CPP, and cannot be used to determine trends or changes in practice over time. Additionally, participant recall bias may have limited the accuracy of findings in this survey, however the limited recall period used (previous eight weeks to three months depending on the question) should have minimized the impact of this.

### Future research

While there are limitations to this study, future research within this topic is warranted. Further research into the perceived effectiveness of TCM treatment from the perspective of women with CPP requires exploration to identify areas relating to women’s self-motivated reasons for TCM use, cost effectiveness, and the experience of care these women encounter. Additionally, investigations into how TCM including acupuncture can fit within the current model of care for women with CPP requires exploration to identify barriers and benefits to interdisciplinary practices including TCM and biomedicine.

## Conclusion

Traditional Chinese Medicine practitioners provide treatments within a biomedical framework, informed by traditional practices and perspectives, and form an important part of a multidisciplinary healthcare team when treating women with CPP. The usage of research to guide clinical treatments was uncommon and may represent barriers in translating clinical trials into clinical practice. Continuity of care and safety could be improved by further integration of TCM into mainstream healthcare services which may be facilitated through improved interprofessional communication.

## Supplementary Information

**Additional file 1.** Survey Tool.

## Data Availability

The datasets used and analysed during the current study are available from the corresponding author on reasonable request.

## Abbreviations

ART: Assisted Reproductive Technologies
CM: Chinese Medicine
CPP: Chronic Pelvic Pain
TCIM: Traditional, Complementary and Integrative Medicine
TCM: Traditional Chinese Medicine
