# Supplementary material for: A cross-sectional study of traditional Chinese medicine practitioner’s knowledge, treatment strategies and integration of practice of chronic pelvic pain in women
Source: BMC Complement Med Ther. 2021 Jun 24;21:174. doi: 10.1186/s12906-021-03355-6 (PMC8229696; doi:10.1186/s12906-021-03355-6)
Supplement: Supplementary file 1 — Additional file 1. Survey Tool. [file 12906_2021_3355_MOESM1_ESM.docx]

**A cross-sectional study of Traditional Chinese Medicine practitioner’s knowledge, treatment strategies and integration of practice of chronic pelvic pain in women**

Susan Arentz^1^, Caroline Smith^1,2^, Rebecca Redmond^3,4^, Jason Abbott^5^, Mike Armour^1,2*^

**Survey Tool**

1. In the past eight weeks, what proportion of your patient/client load have been women?

- All of my patients/clients (I only see women)
- Most of my patients/clients (75% or more)
- About half to three quarters of my patients/clients (50-75%)
- Between a quarter and a half of my patients/clients (25-50%)
- Less than a quarter but I do see women occasionally (<25%)
- I never see female patients/clients

For checked response to ‘I never see female patients/clients’

‘Thank you no further questions.’

2. In the past eight weeks, how often have you seen women wanting treatment for chronic pelvic pain?

- More than once per clinic practice day
- At least every clinic practice day
- Approximately every second clinic practice day
- Approximately every three to seven clinic practice days
- Approximately four occasions during the past eight weeks
- Approximately twice during the past eight weeks
- Once during the past eight weeks
- Women wanting treatment for chronic pelvic pain or associated symptoms rarely present at my clinic

3. During the past eight weeks, what proportion of your female clients have what you would consider abnormal menstrual symptoms based on your Chinese medicine knowledge?

- All my female patients/clients
- Most of my female patients/clients (75% or more)
- About half to three quarters of my female patients/clients (50-75%)
- Between a quarter and a half of my female patients/clients (25-50%)
- Less than a quarter of my female patients/clients (<25%)

4. During the past eight weeks, what proportion of your female clients presented with dysmenorrhea (period pain)?

- All my female patients/clients
- Most of my female patients/clients (75% or more)
- About half to three quarters of my female patients/clients (50-75%)
- Between a quarter and a half of my female patients/clients (25-50%)
- Less than a quarter of my female patients/clients (<25%)

5. During the past eight weeks, what proportion of your female clients presented with dyspareunia (pain during sexual intercourse)?

- All my female patients/clients
- Most of my female patients/clients (75% or more)
- About half to three quarters of my female patients/clients (50-75%)
- Between a quarter and a half of my female patients/clients (25-50%)
- Less than a quarter of my female patients/clients (<25%)

6. During the past eight weeks, what proportion of your female clients presented with dyschezia (pain on bowel motions) or dysuria (pain on urination)?

- All my female patients/clients
- Most of my female patients/clients (75% or more)
- About half to three quarters of my female patients/clients (50-75%)
- Between a quarter and a half of my female patients/clients (25-50%)
- Less than a quarter of my female patients/clients (<25%)

7. During the past 8 weeks how often have women presented with pain related low quality of life or increased absenteeism at work or usual activities due to pain?

- All my female patients/clients
- Most of my female patients/clients (75% or more)
- About half to three quarters of my female patients/clients (50-75%)
- Between a quarter and a half of my female patients/clients (25-50%)
- Less than a quarter of my female patients/clients (<25%)

## Now we want to ask you some questions about your understanding and clinical treatment of women with chronic pelvic pain.

8. Generally speaking what type of treatment and frequency of treatment do you recommend for treatment of chronic pelvic pain? (please select as many as needed)

- Acupuncture more than twice per week
- Acupuncture twice per week
- Acupuncture once per week
- Acupuncture once every two weeks
- Acupuncture once every three weeks or less
- Traditional Chinese herbal medicines (raw and boiled)
- Chinese Herbal granules
- Chinese herbal patent medicines
- Traditional Chinese topical applications (foot baths, creams etc)
- Chinese massage
- Moxibustion
- Acupressure
- Specific traditional Chinese exercises or physical activity
- Dietary changes
- Meditation
- Nutritional supplements

Other (please specify)……………………………………….

9. Generally speaking how frequently do you review your treatment protocol of women with chronic pelvic pain?

- Weekly
- Every two weeks
- Every three weeks
- Every four weeks
- Every month
- Every second month
- Every six months
- Every year
- Every menstrual cycle
- Every second menstrual cycle
- Every third menstrual cycle

Other (please specify)…………………………………………………………………………….

10. In your understanding of chronic pelvic pain in women, how do you assess how severe the women’s pain is during your first consultation? (please select as many as needed)

- By asking and taking notes (or complete a questionnaire) during the consultation (qualitative assessment)
- By using a questionnaire for the client to complete prior to the consultation
- By asking the client to rate their pain on a scale (for example 1-10)
- Assessing the degree of impact on the woman’s usual activities (eg number of days off work)
- Asking about the need to use pain relieving medication
- Using traditional Chinese medicine techniques (tongue and pulse diagnosis)
- Pathology reports such as ultrasound scans
- I don’t measure (or quantify) my clients degree of pain

Other (briefly explain)..................................................................

11. In your understanding of chronic pelvic pain in women, how do you assess the degree of effectiveness of your treatments for pelvic pain as treatment progresses? (Please select as many as appropriate)

- By clients returning for ongoing treatment
- By a questionnaire that the client completes
- Asking the client to rate their pain on a scale (for example 1-10)
- Assessing the impact on the woman’s usual activities (for example number of days off work)
- Asking about the amount of pain relieving medication
- Using traditional Chinese medicine techniques such as changes in the tongue or pulse
- Objective changes in blood markers or scans
- I don’t assess the degree of effectiveness specifically for pain

Other (briefly explain)..................................................................

12. Have you found Chinese medicine practices effective in the management of women with chronic pelvic pain or related symptoms?

- Reducing pain symptoms
- Reducing dysmenorrhoea
- Reducing dyspareunia (pain during sexual intercourse)
- Reducing dyschezia (pain on bowel motions) or dysuria (pain on urination)
- Improving well-being
- Reducing depression and anxiety
- Reducing premenstrual syndrome
- Improving general health
- Improving fertility
- Improving sleep
- Improving bowel function (less constipation or diarrhoea)
- Reducing bloating
- Not sure

Other (please specify)..................................................................................................................

13. How many treatments do your patients/clients require before their chronic pelvic pain or associated symptoms are noticeably reduced using the modalities you selected in Q7? (Please select one)

- 1-3 treatments
- 4-7 treatments
- 8-12 treatments
- 13-20 treatments
- More than 20 treatments are required
- My treatments rarely lead to a noticeable reduction in chronic pelvic pain or associated symptoms
- My treatments do not reduce women’s chronic pelvic pain or associated symptoms

14. What do you think are the main advantages of using Chinese medicine for women with chronic pelvic pain?

- More natural
- Lack of side effects (such as other unwanted effects)
- Effective, it seems to work
- Long lasting effects
- Holistic effects (Improves more than just pain)
- Not addictive
- Can complement existing health care
- Cannot cause harm (cause adverse reactions)

Other......................................................................................................................................

15. What do you think are the main disadvantages of using Chinese medicine in the treatment of women with chronic pelvic pain?

- No disadvantages that I can think of
- Expensive
- Takes longer to work
- Lack of research on whether or not it is effective
- Lack of research on safety such as no warnings
- Not sure if it will work
- Not enough information
- Taste or smell
- Inconvenience of frequent visits
- Not confident to use in conjunction with medical drugs
- Don’t know

Other please specify...............................................

16. If you provide herbal remedies or other ingestible Chinese medicines, how do you check for interactions with either western medicines or other complementary therapies or medicines the woman may be taking? (Select the most common answer)

- Ask women directly what they are taking as part of the consultation
- Rely on patients/clients to tell me if they are on medication/supplements
- Rely on details in referral letters from other practitioners
- I don’t ask women what they are taking

Other (please specify)………………………………….

17. Has any women that you have treated for chronic pelvic pain reported a negative reaction to your treatment?

- Yes
- No (please go to question 18)

*Drop down menu for a Yes response:*

17. A. If yes which type of Chinese medicine?

- Chinese herbs (raw herbs boiled)
- Chinese herbs (granules)
- Acupuncture
- Moxibustion
- Traditional Chinese herbal tablets or teas

Other...............................................................................................................................

17. B. Did the adverse effect:

- Fully resolve following the treatment with no reaction to subsequent treatment
- Fully resolve but return following subsequent treatments
- Partially resolve
- No resolution and adverse effect continued

Other (Please explain)………………………………………………………………….

### Now we would like to ask you some questions about your sources of information and your referral networks

18. During the past eight weeks, how many women have been referred to you from other health or medical providers?

- Zero
- One to three
- Four to six
- Seven or more

Other (please specify)………………………………………………………………….

*Drop down menu for answers other than Zero*

18.A. Which type of complementary medicine, allied health or medical provider referred to you?

- Exercise physiologist
- Physiotherapist (General)
- Pelvic physiotherapist
- Podiatrist
- Other Chinese medicine practitioner
- Naturopath
- Western Herbal medicine practitioner
- Homeopath
- Reflexologist
- Osteopath
- Chiropractor
- Massage therapist
- Pharmacist
- General practitioner
- Gynaecologist
- Endocrinologist

Other (please specify)..............................................................................................

18B. Do referred clients present with a letter of introduction explaining the reason for their referral to you?

- Yes
- No
- Sometimes

Other (please specify)…………………………………………………..

19. During the past eight weeks, how many times have you referred women clients to other health or medical professionals?

- Zero
- One to three
- Four to six
- Seven or more

Other (please specify)………………………………………………………………….

*Drop down menu for answers other than Zero*

19A. Which types of health or medical practitioners do you refer women with pelvic pain? (Please choose as many as you wish)

- Exercise physiologist
- Physiotherapist (general)
- Pelvic physiotherapist
- Podiatrist
- Other Chinese medicine practitioner
- Naturopath
- Western herbal medicine practitioner
- Homeopath
- Reflexologist
- Osteopath
- Chiropractor
- Massage therapist
- Pharmacist
- General practitioner
- Gynaecologist
- Endocrinologist

Other (please specify)..............................................................................................

19B. Do you usually write a letter of introduction to the doctor (email or phone call) outlining the reason for referral of your client?

- Yes
- No
- Sometimes

Other (please specify)……………………….

*If yes was answered the following drop down question:*

20. Do you refer women directly to pathology collection for blood tests or other pathology tests?

- Yes
- No
- Sometimes

Other (please specify)……………………….

21. In your understanding of chronic pelvic pain, what pathology do you think may be associated? (Please select as many as appropriate)

- Lower back structural problems such as a bulging disc
- Polycystic ovary syndrome
- Endometriosis
- Fibroids
- Adenomyosis
- Inflammatory bowel diseases
- Urinary tract infections

Other (please explain)…………………………………………………………………………………….

22. What western medical and/ or Chinese medicine conditions do you perceive may be the underlying drivers or causes of chronic pelvic pain? (please select as many as appropriate)

- Inflammation
- Muscle spasm
- Not known
- TCM pattern – Blood stasis
- TCM pattern – Qi stagnation
- TCM pattern – Cold stagnation
- TCM pattern – Yang deficiency
- TCM pattern – Damp Phlegm.
- TCM pattern - Other

Others (please specify)……………………………..

23. What guides your treatment of chronic pelvic pain? (e.g  point selection, selection of herbal medicines etc.)

- The Chinese medicine diagnosis ONLY
- The western medicine diagnosis ONLY
- A combination of western and Chinese medicine diagnosis
- Discussion with clinical peers
- Text books and lecture notes
- Articles from peer reviewed journals
- Updates from online sources

Other (please specify)……………………………………….

24. What are the main sources of information you rely on to develop to keep up to date on how to treat women with chronic pelvic pain? (please tick as many as appropriate)

- Traditional Chinese medicine understanding from textbooks
- Traditional Chinese medicine understanding from teachers during training
- Western medical information from official sources (such as NIH)
- Short seminars and events
- Professional association events and conferences
- Australian Government (Therapeutic Goods Administration)
- Materials on the internet (such as forums or blogs)
- On-line courses (e.g Pro-D seminars)
- Referring to articles in peer reviewed journals
- Other (please specify)………………………………………………………

## Finally some questions about you, to help us describe the Chinese Medicine practitioners who have taken part in the survey.

25. Are you aged between:

- 18-24
- 25-29
- 30-34
- 35-40
- 40-44
- 45+

26. Please identify your gender

- Female
- Male
- Intersex
- Prefer not to answer

27. In which country did you study Chinese Medicine?

- Australia
- China

Other please specify...........................................................................................

28. Which of the following best describes your Chinese Medicine practice? (tick as many as required)

- Employed in clinical practice
- Self-employed in clinical practice
- Enrolled in further study
- Having a break from clinical practice
- Informal treatment of family and friends

Other............................................................................................................................................

29. How much time per week do you practice?

- 1 day per week
- 2-3 days per week
- 4-5 days per week
- 6-7 days per week

30. Do you offer private health insurance rebates?

- Yes
- No

31. How many years have you been in Chinese medicine practice?

- Less than a year
- One to three years
- Three to six years
- Six to ten years
- Ten to fifteen years
- More than fifteen years

32. What state in Australia do you live in?

(Drop down list)

- NSW
- SA
- ACT
- WA
- VIC

33. Do you live in an urban, rural or remote area?

(Drop down)

- Urban
- Rural
- Remote

Thank you very much for your help. Please remember to click on the submit button at the bottom of this page.

If you are interested in participating in more research exploring Chinese Medicine practitioner’s treatment of women with chronic pelvic pain, please click on the link and provide your contact details. You will be contacted by the researchers and provided with a participant information sheet for your consideration.

Name:

Contact email address:

Do you live in the greater Sydney area? Y/N

What is the postcode of your clinical practice?

Thank you
